# Personalized Medicine in Ophthalmic Diseases: Challenges and Opportunities

**DOI:** 10.3390/jpm13060893

**Published:** 2023-05-25

**Authors:** Kai Jin, Chun Zhang

**Affiliations:** 1 Eye Center, The Second Affiliated Hospital, School of Medicine, Zhejiang University, Hangzhou 310009, China; 2 Department of Ophthalmology, Peking University Third Hospital, Beijing 100191, China

## 1. Introduction 

Personalized medicine is a broadly used term to encompass approaches used to tailor healthcare to the needs of individual patients [1]. It can lead to more effective treatments for patients in ophthalmology, reducing the need for trial-and-error approaches and potentially avoiding unnecessary treatments. This can also lead to cost savings for healthcare systems. Diagnostic techniques that can realize comprehensive individual assessment are important. Next-generation sequencing and translational research are some of the techniques put forward by previous studies. Gene therapy-based treatment trials have been presented for ophthalmic diseases, such as retinitis pigmentosa and age-related macular degeneration.

## 2. The Role of Artificial Intelligence and Telemedicine in Diagnostic Techniques

Recently, with the rapid development of Artificial Intelligence and interdisciplinary collaboration, concepts like machine learning and wearable device have been frequently raised in ophthalmic research [2]. There might be new promising methods to realize personalized ophthalmology. The ophthalmology field was among the first to adopt Artificial Intelligence (AI) in medicine. The availability of digitized ocular images and substantial data have made deep learning (DL) a popular topic. At the moment, AI in ophthalmology is mostly used to improve disease diagnosis and assist decision-making in ophthalmic diseases such as diabetic retinopathy (DR), glaucoma, age-related macular degeneration (AMD), cataract and other anterior segment diseases. However, most of the AI systems developed to date are still in the experimental stages, with only a few having achieved clinical applications. There are a number of reasons for this phenomenon, including security, privacy, poor pervasiveness, trust and explainability concerns [3]. Telemedicine screening needs to be tailored to the targeted population in order to reap the benefits of digital technology.

## 3. Gene Therapy-Based Treatment and Personalized Medicine

Recent developments in the field of gene therapy have attracted interest from scientists, clinicians and industry. Gene therapy approaches with the most promise in terms of visual improvements and longevity can be determined with the help of retinal and deep phenotyping. Progress in genotyping techniques and back-of-the-eye scans are helping us understand the diseases and their manifestations in patients. The majority of vision loss in diseases of the eye is caused by the loss of photoreceptor function. The appropriate therapeutic approach to use for each patient is determined by the timing and circumstances surrounding the loss of photoreceptor function. Gene therapy is rapidly becoming a therapeutic reality in the clinic. The move from laboratory work to clinical application has been propelled by advances in our understanding of disease genetics and mechanisms [4]. The beginning of the twenty-first century was marked by the innovative use of pharmacochemical interventions. One of the first applications of novel genome editing technologies was the treatment of rare inherited retinopathies. A new era of precision medicine will be ushered in by the exciting development of newer, cutting-edge strategies including base editing and prime editing [5].

## 4. Challenges and Opportunities 

AI coupled with teleophthalmology presents an opportunity to promote equity in eye health [6]. It is indicated that novel screening strategies, such as AI-based screening, could achieve greater cost-effectiveness in population screening. Routine screening for multiple blindness-causing eye diseases could be highly cost-effective in China, providing robust economic evidence for informed policy making regarding its large-scale promotion. Although ophthalmic AI and telemedicine show promise for patients, there are significant barriers to widespread adoption. Clinicians will be tasked with embracing innovation while ensuring protocols and implementation are evidence-based and improve outcomes [7]. Interpretability and expandability are crucial factors in AI-based medical screening systems. Interpretability refers to the ability of the system to provide clear and understandable explanations for its decisions, which is important for gaining the trust of medical professionals and patients. Expandability refers to the ability of the system to adapt and improve over time as new data become available [8].

The first successful implementation of AAV-mediated gene augmentation therapy is for the treatment of retinitis pigmentosa, a dozen other clinical trials are underway to tackle other monogenic diseases of the retina using this strategy [4]. Gene therapy still has a negative effect on the eye. Different cell types require different gene therapies. The cost of clinical trials will likely be reduced in the years to come as a result of this and anticipated developments in the manufacturing practices of core technologies. In the years to come, methods to safely and specifically edit the genes are likely to be crucial. Due to the complexity of silence and replacement strategies, clinical trials have not been able to target the autosomal-dominant genes causing retinal degeneration. Future developments in the evaluation of low vision along with development of more sophisticated instruments for objective measures is going to be key to the achievement of such therapies.
